# Removing information from working memory with a delay: Effective but not beneficial

**DOI:** 10.3758/s13423-024-02550-z

**Published:** 2024-08-05

**Authors:** Hannah Dames, Chenyu Li, Gidon T. Frischkorn, Klaus Oberauer

**Affiliations:** Department of Psychology, University of Zurich, Binzmühlestrasse 14/22, 8050 Zurich, Switzerland

**Keywords:** Working memory, Removal, Updating, Directed forgetting

## Abstract

Ideally, removing outdated information from working memory (WM) should have two consequences: The removed content should be less accessible (*removal costs*), and other WM content should benefit from the freeing up of WM capacity (*removal benefits*). Robust removal benefits and removal costs have been demonstrated when people are told to forget items shortly after they were encoded (immediate removal). However, other studies suggest that people might be unable to selectively remove items from an already encoded set of items (delayed removal). In two experiments (*n* = 219; *n* = 241), we investigated the effectiveness and consequences of delayed removal by combining a modified version of Ecker’s et al. (*Journal of Memory and Language, 74,* 77–90, 2014) letter updating task with a directed-forgetting in WM paradigm. We found that while delayed removal resulted in reduced memory for the to-be-forgotten item-location relations (removal costs), it failed to enhance performance for existing WM content. This contrasts sharply with immediate removal, where removal benefits can be observed. A fine-grained analysis of removal benefits shows that removal from WM proactively facilitates the subsequent encoding of new information but does not retroactively aid stored WM content.

## Introduction

Working memory (WM), which holds mental representations temporarily available for processing or action (Oberauer et al., 2018), has severely limited capacity. Because of our ever-changing environment and the corresponding frequent shifts in our thoughts, the contents of WM must be constantly updated. WM updating has been described as a combination of two processes: (1) the removal of information from WM and (2) the encoding of new information in its place. The *removal* of outdated information from WM should have two consequences (Lewis-Peacock et al., 2018). First, the removed content should be less accessible (*removal costs*). Second, freeing WM capacity for relevant information should benefit remaining WM contents (*removal benefits*).

A recent study (Dames & Oberauer, 2022) provided evidence for both these predictions. In a series of four experiments, participants were instructed to remember up to six words. After the offset of each word, a cue indicated whether this word should be remembered or could be forgotten. Additionally, there were two baseline conditions in which all words had to be remembered. In the *large set-size* baseline, all six presented words were to be remembered. In the *small set-size* baseline only three (± 1) words were presented, all of which were to be remembered. In the removal condition, three (± 1) out of six words were cued to be forgotten, so that the number of remaining to-be-remembered words was equal to the small set-size baseline. Dames and Oberauer (2022) found that performance for the remaining to-be-remembered words in the removal condition was as good as for the small baseline condition, and much better than when all six words had to-be-remembered (i.e., the large baseline condition). This finding demonstrates removal benefits: After removing to-be-forgotten items, memory for the remaining items was as good as if the to-be-forgotten items had never been presented.

On a small subset of trials, one of the to-be-forgotten words was probed—on all other trials, only to-be-remembered words were tested. For to-be-forgotten words, memory performance was much poorer than for to-be-remembered words. This finding shows the removal cost. Together, consistently poor memory for to-be-forgotten words and facilitation of to-be-remembered words demonstrated that people can remove outdated information from WM.

**Fig. 1:**
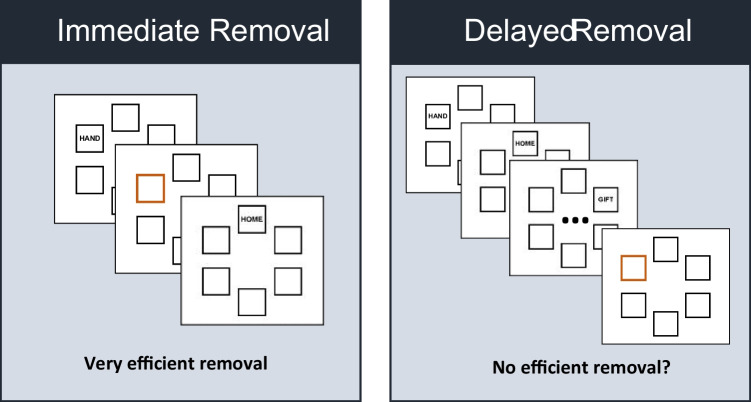
Difference between immediate and delayed removal. *Note.* In immediate removal, presentation of each item is followed by a cue to maintain or forget the item before further items are presented. In the example display, the orange box frame instructs participants to forget the word that was presented at that location. In delayed removal, all items of the memory set are presented and encoded, and then a subset is cued as to-be-removed

**Fig. 2:**
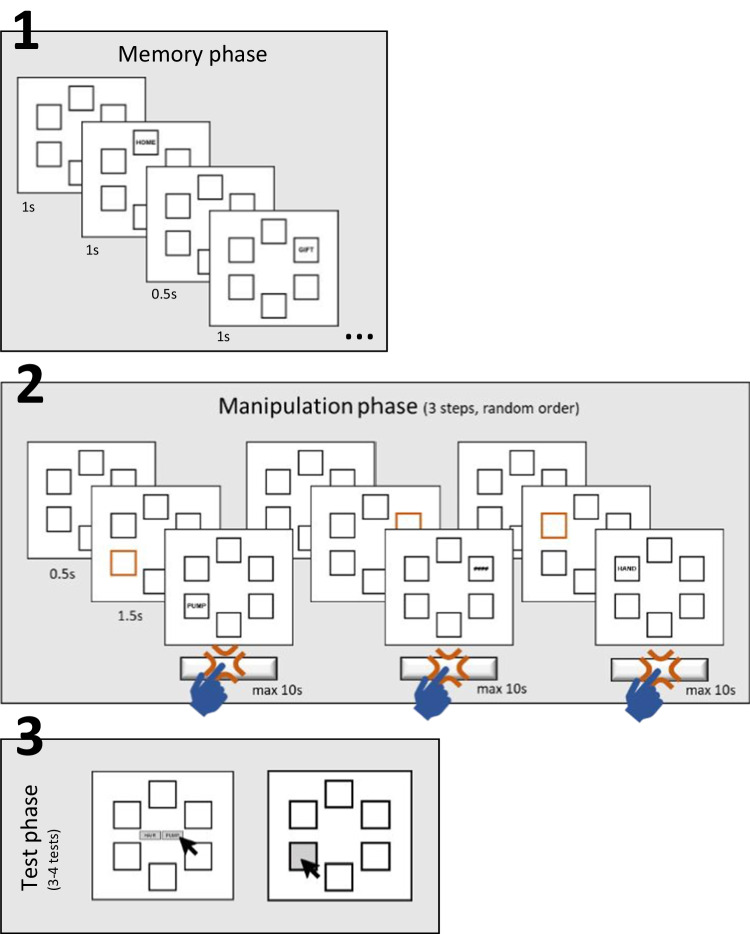
General trial structure of Experiment 1

Because in Dames and Oberauer (2022) removal (or remember) cues were presented immediately after the offset of each word, participants always removed items from WM that were most recently encoded. We term this kind of removal *immediate removal* (see Fig. 1, left side). In the current study, we explore whether people can also remove information from WM that is not the last information they have encoded (*delayed removal*), such as when being asked to forget a particular word from an already fully encoded set of words (see Fig. 2, right side).

There is good reason to question the efficiency of delayed removal in comparison to immediate removal. Previous research by Oberauer (2018) employed a paradigm akin to that of Dames and Oberauer (2022), except that removal cues were given only after the complete memory set had been encoded. These cues did not yield a removal benefit. Memory accuracy for the to-be-remembered information in the removal condition, in which three of the six words were cued to be removed, was not better than that of the large set-size baseline in which all six words had to be remembered. Because Oberauer (2018) never tested memory strength of the to-be-forgotten words directly, his findings are compatible with two possibilities: (1) People might be unable to selectively eliminate information from an already encoded memory list in WM, or (2) participants were able to remove information, but doing so did not benefit the already encoded items in WM.

In the current study, we aim to decide between these two possibilities. By occasionally probing memory for a to-be-removed item, we test whether people are able to remove specific items from an already encoded set of items (delayed removal). Further, by examining the differences between immediate and delayed removal, we aim to precisely determine the conditions under which removal benefits are obtained.

## Experiment 1

We first aimed to test the existence of removal costs and benefits for delayed removal. For this, we asked participants to remember six words presented one-by-one across six boxes. Then, participants worked through three removal or updating steps. In each of these steps, one of the six boxes was cued, indicating that the word in that box should be forgotten. Following the cue, either a new word was presented (updating condition) or “####” was presented, signaling that no new word had to be encoded but the old word could still be forgotten (removal condition). We mixed removal steps with updating steps so that people would be more motivated to remove the cued item when they expected that they might have to replace it with a new item.

After participants completed all three steps, we tested their memory for a subset of the words. In most of the trials, we tested participants’ memory for the to-be-remembered words (either for words that had not been cued, or for newly encoded words in updating boxes). On a small subset of trials, we tested participant’s memory for a to-be-forgotten word from a removal-only box (in which “####” was presented). We did this only rarely to avoid creating an incentive for trying to remember to-be-removed items.

Occasionally testing the to-be forgotten words enabled us to test removal costs by assessing to what extent participants are still able to remember the last contents of “####” boxes or not. Additionally, by asking participants to forget some information without encoding new information (removal condition), we tested whether memory performance improved when the final memory set size decreased by removing items without replacing them with new stimuli, thereby assessing removal benefits: If removal was successful, it should reduce the set size at the time of test to 6-X, where X is the number of to-be-forgotten items. To test this, we analyzed whether memory for the to-be-remembered items increased with the number of to-be-forgotten items, demonstrating removal benefits (Dames & Oberauer, 2022; Oberauer, 2018).

## Method

Methods, hypotheses, and the data analysis procedures were preregistered and can be found online (https://osf.io/ae632). The preregistration also provides more detailed information on the randomization procedure.

### Participants and exclusion criteria

In total, 302 native English speakers were recruited via the web-based participation platform Prolific for participation in the online experiment. To ensure high data quality, we instructed participants not to leave their browser window while completing the memory task. If they did so more than twice, the experiment automatically aborted, and no data were collected. To ensure that participants paid attention to our instructions, we excluded participants who failed two attentions checks during the instructions (no data were collected).

After applying a list of preregistered exclusion criteria, the final sample consisted of 219 participants (*M*_age_ = 27.6 years, *SD*_age_ = 4.5, range: 18–35 years; 57.1% male, 41.1% female, 1.8% other): We excluded participants who stated that they did not participate seriously in the experiment, or did not agree that their data could be used (*n* = 2), who had technical issues during completion (*n* = 2), who indicated that they used paper and pen to remember the words (*n* = 2), and who had a mean error rate (old vs. new selection) of higher than 30% for responses to no-change boxes in set-size 6–3 trials (*n* = 15; see below for a description of the different set-size conditions). Additionally, we excluded 62 participants who did not follow or misunderstood the instructions (i.e., stated that they tried to remember the word originally presented in a box upon a “####”; see Experiment 2 for a solution to this issue).

We collected a large sample because we were interested in memory performance of to-be-forgotten items, which we tested only in three trials per participant within the entire experiment. The sample size was chosen based on prior experiments (Dames & Oberauer, 2022). As we used Bayesian analyses, we planned to increase the sample size if we found that some hypothesis tests were ambiguous (i.e., Bayes factors between 1/3 and 3; this criterion was preregistered). This turned out not to be necessary.

We only recruited participants with normal or corrected-to-normal vision who did not participate in our previous studies in which we tested to-be-forgotten information. Participants signed an informed consent form prior to the study, and they were debriefed at the end. The study protocol is in line with the guidelines of the institutional ethics review board.

### Material

Frequent English nouns with a length of four or five letters served as stimuli. For every participant, words were randomly drawn from a pool of 600 frequent words (mean Kucera-Francis written frequency = 79.2). A list of all word stimuli can be found online within our OSF repository. The experiment was programmed using jsPsych (de Leeuw, 2015; Kuroki, 2021).

### Trial structure

The structure of the experiment is illustrated in Fig. 2. A given trial consisted of three phases: the encoding, the removal-updating, and the test phase.

**Encoding phase.** Participants first saw six rectangular boxes arranged in a circle (60° apart from each other). Box frames had thin black outlines and were presented on a white background. In the encoding phase, after presentation of all six empty boxes for 1 s, six words were displayed one by one for 1-s each in a clockwise order starting in the left top box at 10 o’clock. Participants were instructed to memorize all words. Following each word offset, all boxes remained empty for 0.5 s before the next word was presented in the next box.

**Removal-updating phase.** After the last word has been presented, a series of updating/removal steps for a random subset of three words followed. Each updating/removal step began with an interval of 0.5 s during which all boxes remained empty. Then the box of one word turned orange for 1.5 s, which served as a cue indicating that this word should be forgotten. After that, either a new word was presented that participants had to remember in place of the old word in that box (updating condition), or “####” was presented signaling that no new word had to be encoded but the old word could still be forgotten (removal condition). The new word or the “####” were presented until participants pressed the space bar to indicate that they had completed the step (i.e., encoding of the new word or the processing of the “####”), with a maximum response time of 10 s. Then the next updating/removal step followed. The distribution of removal versus updating steps over the three steps of a trial differed depending on the set-size condition (see below).

**Test phase.** After all updating/removal steps of a trial were completed, participants’ memory for up to four (usually, to-be-remembered) words was tested.

There were three different test types: (1) Tests of *no-change boxes*—In that test type, the word was presented during the encoding phase, did not receive any cue, and did not undergo the updating process. The to-be-remembered word was tested. (2) Tests of *updating boxes*–The word in this box was cued and followed by a new word that was to be remembered; the new to-be-remembered word was tested. (3) Tests of *removal boxes*—The word in that box was cued but no new word had to be remembered (“####” has been presented). Accordingly, a to-be-forgotten word (followed by the “####”) was tested. Participants were instructed that in this case they should try to remember the word that had originally been presented in that box.

We assessed participants’ item and binding memory in form of a two-step test: First, we tested item memory (i.e., memory for which items had been presented in the current trial) by presenting one to-be-remembered word together with a completely new word in the center of the screen. Using their computer mouse, participants selected the word that was previously presented in that trial. Next, to measure binding memory (i.e., memory of the relation between an item and its location), we asked participants to select the box in which the selected word was previously presented by clicking on it. In the majority of trials (approximately 94%), only boxes still containing a to-be-remembered word—updating boxes and no-change boxes—were tested in random order. On three trials, we tested participants’ item and binding memory for to-be-removed words belonging to “####” boxes. In these trials, for the item memory test, instead of a to-be-remembered word, a to-be-forgotten word of that trial was presented together with a new word.

**Set-size conditions.** There were four set-size conditions described by the number of initially encoded words (always 6) minus the number of to-be-forgotten words: set-sizes 6–0, 6–1, 6–2, and 6–3. In the set-size 6–0 condition, during the updating/removal phase, participants went through three updating steps (three new words replaced the old ones) and no pure removal steps. In contrast, in the set-size 6–3 condition, during the updating/removal phase, participants could forget three words (replaced by ####) and thus went through no updating steps. In the remaining set-size 6-X conditions, out of the six words, participants could forget X words without a replacement, and had to update the remaining 3-X words that were cued. Across the whole experiment, there were 12 trials per set-size condition (hence 48 trials in total). For set-size 6–0 trials (0 removal-only + 3 updating boxes), we tested two random no-change boxes and two random updating boxes. For set-size 6–1 trials (1 removal-only + 2 updating boxes), we tested two random no-change and both updating boxes. For set-size 6–2 trials (2 removal-only + 1 updating box), we tested three random no-change and one updating box. For set-size 6–3 trials (3 removal-only + 0 updating boxes), we tested the three no-change boxes in random order.

In addition, there were three trials where we tested participants’ memory for the to-be-forgotten words in removal-only boxes (one trial with 2 removal-only boxes, one trial with 3 removal-only boxes; and one trial with either 2 or 3 removal-only boxes, chosen at random). These trials were randomly assigned to either the first (Trial 16), second (Trial 32), or third (Trial 48) position. To reduce the chance that the trial with to-be-forgotten tests can be predicted, these trial boxes were shuffled by adding ± 1 trial at random. Thus, in total there were 51 trials in the main part of the experiment.

### Procedure and instructions

After providing informed consent, instructions were given on-screen. The instructions explained the general procedure and the updating steps (see above). In the updating condition, we instructed participants to press the space bar as soon as they have remembered each new word and its spatial position. In the removal condition, we instructed participants to press the space bar as soon as they have forgotten the word in the box marked with “####.” We told participants to remember only the to-be-remembered words (learned during the encoding phase, or the newly updated words) for a subsequent memory test, and that they should try to forget the cued to-be-forgotten words. We informed participants that on very few occasions their memory for to-be-forgotten words followed by a “####” would be tested. In such cases, participants were instructed to do their best to remember the to-be-forgotten words and their location. We told participants to nevertheless truly try to forget all items that are cued to be forgotten, as this would make it potentially easier for them to remember the to-be-remembered items. This is the same procedure that was used in previous experiments investigating immediate removal (Dames & Oberauer, 2022), and which led to a substantial removal cost when the to-be-forgotten items were tested. Participants were instructed not to use any external help (e.g., use pen and paper) and to respond as quickly and accurately as possible.

Following the instructions, we tested participants’ understanding of the task instructions. If participants failed one question during this comprehension test, they had to re-read the instructions. Next, participants practiced their task on four trials. In the practice block, participants received feedback (“correct”/ “incorrect”) immediately after their responses. They received no feedback on their performance for the remainder of the experiment. After the practice block, participants had the option to reread the instructions and redo the practice block, or proceed with the experiment. Next, the main experimental block (51 trials) with the opportunity for short breaks after trial 20 and 40 followed.

Using a postexperimental questionnaire, we checked whether participants had any suspicion about the purpose of the experiment. We took special care to additionally check whether participants correctly understood the task by asking them about their comprehension of the instructions and the task. Specifically, we asked what they were supposed to do and what they actually did when they saw the cue, and when they saw the “####.” Participants were also asked to describe what they thought they were supposed to do when to-be-forgotten words were tested, and if they tried to respond correctly on those trials. We additionally assessed how they responded on to-be-forgotten tests using a multiple-choice question. Last, we asked them about their strategies to remember, forget, or update the words/boxes and whether they used external help to remember the words. Participants were debriefed about the reason behind testing to-be-forgotten stimuli.

### Data analysis

We analyzed the accuracy of responses to individual trials using Bayesian generalized linear mixed models (BGLMMs) assuming a Bernoulli data distribution predicted by a linear model using a logistic link function. We estimated these models with the R package brms (Bürkner, 2017). All our models included random intercepts for participants. For the regression coefficients, we used moderately informative normal priors with a mean of 0 and a standard deviation (*SD*) of 1.^1^ For the standard deviations of random effects, we used weakly informative priors on the positive real line (half student *t*-prior with three degrees of freedom and a scaling parameter of 2.5). All categorical predictors were coded as sum-to-zero contrasts, and continuous predictors were mean-centered and normalized so that their *SD* = 1. We estimated the posteriors by sampling parameter values using the No-U-Turn Sampler (NUTS; an extension of the Hamilton Monte Carlo sampling method) as implemented in Stan (Carpenter et al., 2017). We sampled four independent Markov chains with 50,000 iterations each (2,000 warm up each). To investigate convergence, we inspected R-hat values (ratio of between-chain variance to within-chain variance). R-hat values were ≤ 1.01 for all parameters in every model.

We computed Bayes factors (estimated through the bridge sampler; Gronau et al., 2017) to estimate the strength of evidence for the null and the alternative hypothesis. To this end, we fitted two competing, nested models for each hypothesis, one including the effect of interest and one that did not. We then compared the two models to calculate the evidence for or against the effect in question. We report Bayes factors in favor of an effect as BF_10_, and Bayes factors in favor of the null hypothesis as BF_10_. We considered BFs larger than 3 to be substantial evidence for one hypothesis over the other. To give an example, a BF_10_ of 3 would indicate that the data are 3 times more likely under the alternative hypothesis than under the null hypothesis.

## Results

**Fig. 3:**
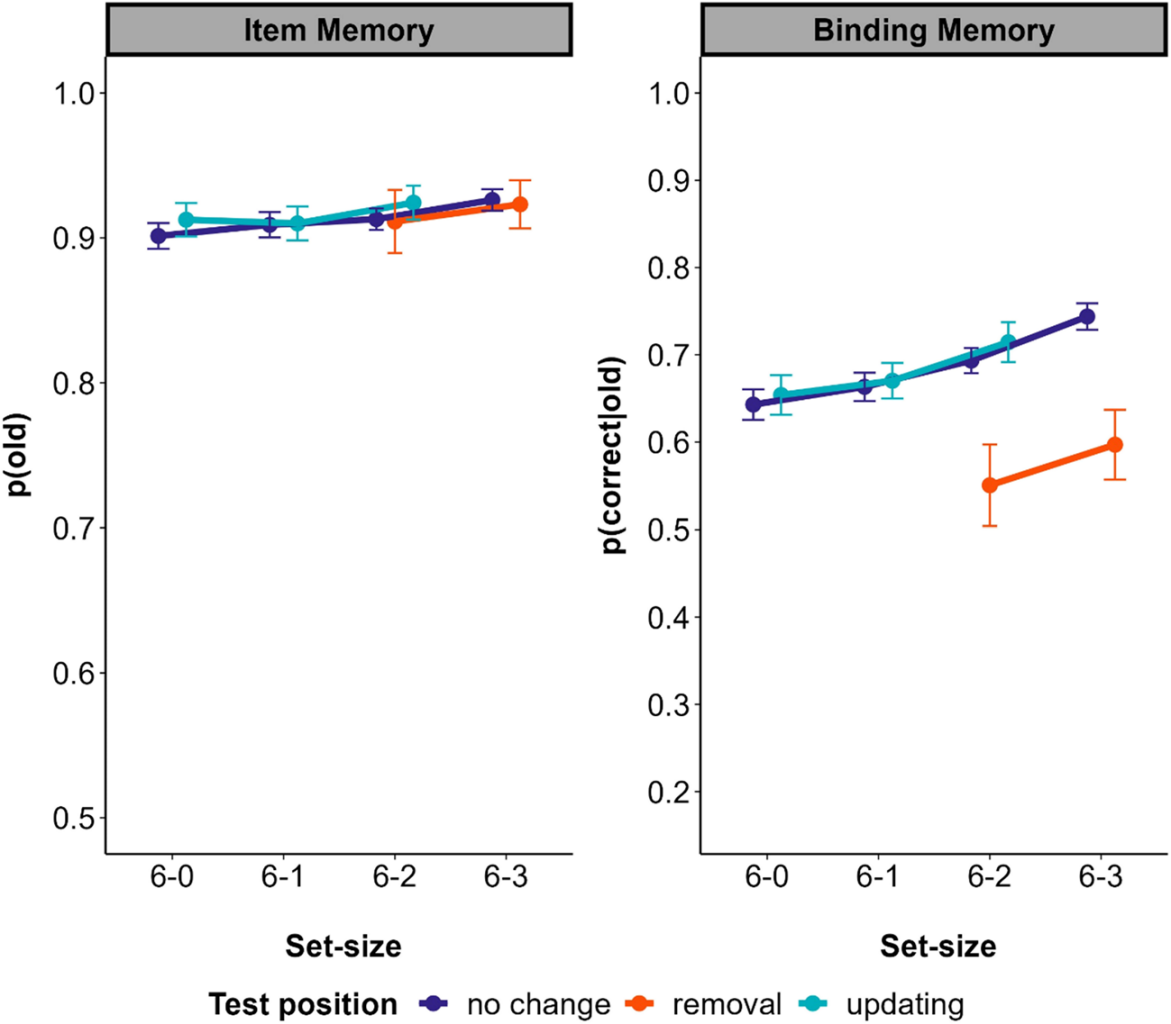
Mean accuracy rates across set sizes and box types in Experiment 1. *Note*. The 6-X conditions describe the number of initially encoded words (always 6) minus the number of to-be-forgotten words. Out of the six words, participants could forget X words without a replacement, and had to update the remaining 3-X words that were cued. Error bars represent the 95% within-subjects confidence intervals. Note the different scales of the *y*-axes, which range from chance to perfect performance for both kinds of memory. (Color figure online)

Figure 3 displays mean accuracy rates as a function of set-size conditions and test types. Results as a function of serial position can be found online (https://osf.io/f68wz/).

### Removal costs

To estimate the availability of to-be-forgotten items, we compared performance of removal and no-change boxes for the set-sizes 6–2 and 6–3. Item memory, measured as the probability to correctly identify the probe over a new word (p(old) was the same for to-be-forgotten removal boxes and to-be remembered no-change boxes (BF_01_ = 20.5; evidence in favor of a model excluding the main effect of condition compared with a model including the effect). Binding memory, measured as the probability to correctly select the correct position a word was presented in, given that participant correctly identified it as old word (p(correct|old)), was substantially lower for the removal boxes than for no change boxes (BF_10_ = 3.4 × 10^19^; evidence in favor of a model including the main effect of box type compared with a model excluding the effect). Thus, for delayed removal, we observed removal costs for binding but not item memory.

### Removal benefits

To estimate removal benefits, we tested whether memory for the to-be-remembered items increased with the number of to-be-removed items (0 to 3). For both item (BF_10_ = 11,713.6) as well as binding (BF_10_ = 2.5 × 10^38^) memory accuracy increased with the number of to-be-forgotten items, demonstrating removal benefits.

## Discussion

Removal costs for to-be-forgotten words in removal boxes showed that these items were less available in WM than to-be-remembered words in no-change boxes. Furthermore, we observed removal benefits, as memory performance increased with the number of to-be-removed items. This finding stands in contrast with the lack of removal benefits in Oberauer (2018). In Experiment 1, however, the number of to-be-forgotten items was confounded with the number of words to be updated. Lower memory performance for set-size 6–0 as compared with set-size 6–3 could be explained by participants having to update several words at set-size 6–0 but none at set-size 6–3. We tested this possibility in Experiment 2.

## Experiment 2

The aim of Experiment 2 was to replicate the findings of Experiment 1 while introducing a baseline condition for a more rigorous test of removal benefits with delayed removal. In Experiment 1, we observed both removal costs and benefits. However, in that experiment, we could not determine whether the benefit of removing several items stems from reduced WM load or from an increase in updating steps. In Experiment 2, to test whether removal of information from WM results in a facilitation effect, on some trials, participants had to remember all six items and did not go through any manipulation steps (no removal or updating steps). This “remember-all” condition served as a baseline. If memory performance in the set-size 6–3 was better than in the new remember-all baseline condition, we could infer that removing information benefitted memory for the remaining still-relevant WM content.

Moreover, in Experiment 1, some participants reported difficulties correctly following the double forget cues: Forgetting (1) when the box frame turned orange, continue forgetting (2) when a “####” appeared. Although we excluded those participants from our analyses, the misunderstanding of instructions in Experiment 1 led to relatively high exclusion rates. In Experiment 2, we made several improvements to our procedure to tackle this issue (see Procedure).

## Method

Methods, hypotheses, and the data analysis procedures were preregistered and can be found online (https://osf.io/e5krc).

### Participants

The same preregistered inclusion and exclusion criteria as in Experiment 1 were applied and we only recruited participants that had not participated in the previous experiment. From the initial sample (*N* = 298), 57 participants had to be excluded, resulting in a final sample size of *n* = 241 participants (*M*_age_ = 27.3 years, *SD* = 4.7, range: 18–35 years; 59.8% male, 38.6% female, 1.7% diverse). Probably due to our improved procedure (see below), only half as many participants (*n* = 37) compared with Experiment 1 had to be excluded because they did not try to forget the to-be-forgotten words without a replacement or misunderstood the task.

### Material and procedure

Stimuli and procedure were identical to Experiment 1 with the following exceptions: First, we introduced a remember-all baseline condition, where, on some trials, no manipulation steps took place. On these trials, all box frames turned blue for 9 s, indicating that all words needed to be remembered. Second, following the instructions, we extended the instruction tests participants had to pass in order to move on with the practice trials (two additional questions). Third, in the manipulation phase, updating and removal steps were no longer self-paced. That is, after the pre-cue was presented for 1.5 s, either a new word was presented (updating condition) for 1 s (box frame remained orange during that time), or all boxes remained empty for a further 1 s, signaling that no new word had to be encoded but the old word could still be forgotten (removal condition). Subsequently the next updating/removal step followed.

Fourth, we reduced the total number of trials from 51 to 43 trials, and after Trial 15 and Trial 30, we gave participants the opportunity for a short break. There were now eight trials for set-sizes 6–0, 6–1, 6–2, 6–3, and eight trials for the remember-all condition (where four random no-change boxes were tested), as well as three additional trials in which we tested participants’ memory for to-be-forgotten words from removal boxes.

### Statistical analyses

The statistical analysis procedures were identical to those of Experiment 1.

## Results

**Fig. 4:**
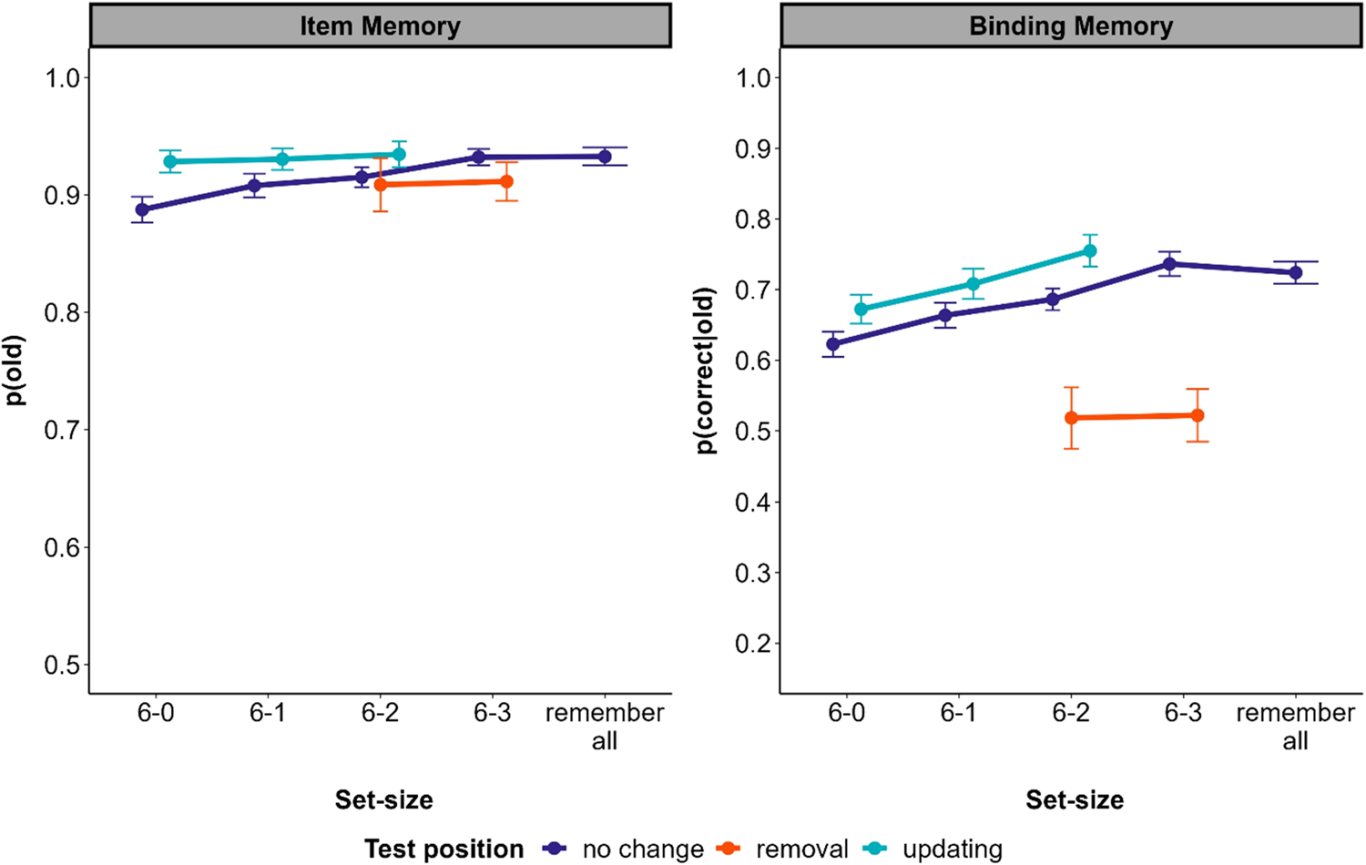
Mean accuracy rates across set sizes and box types in Experiment 2. *Note*. The 6-X conditions describe the number of initially encoded words (always 6) minus the number of to-be-forgotten words. Out of the six words, participants could forget X words without a replacement, and had to update the remaining 3-X words that were cued. Error bars represent the 95% within-subjects confidence intervals. Note the different scales of the *y*-axes, which range from chance to perfect performance for both kinds of memory. (Color figure online)

Mean accuracy rates for the different set size conditions and test types are displayed in Fig. 4.

### Removal costs

Replicating the results of Experiment 1, binding memory (BF_10_ = 2.1 × 10^43^) but not item memory (BF_01_ = 13.9) was substantially lower for removal than for no-change boxes.

### Removal benefits

As in Experiment 1, memory for items (BF_10_ = 6.2 × 10^11^) as well as bindings (BF_10_ = 4.9 × 10^32^) increased with the number of to-be-forgotten items. However, for the critical comparison between set-size 6–3 and the remember-all condition, we observed no performance difference, neither for item (BF_01_ = 26.4) nor for binding (BF_01_ = 10.2) memory.

Thereby, we could show that people are able to remove information (i.e., the relation between item and location) from an already encoded set of items, but this delayed removal does not facilitate memory for the remaining WM content.

## General discussion

**Fig. 5:**
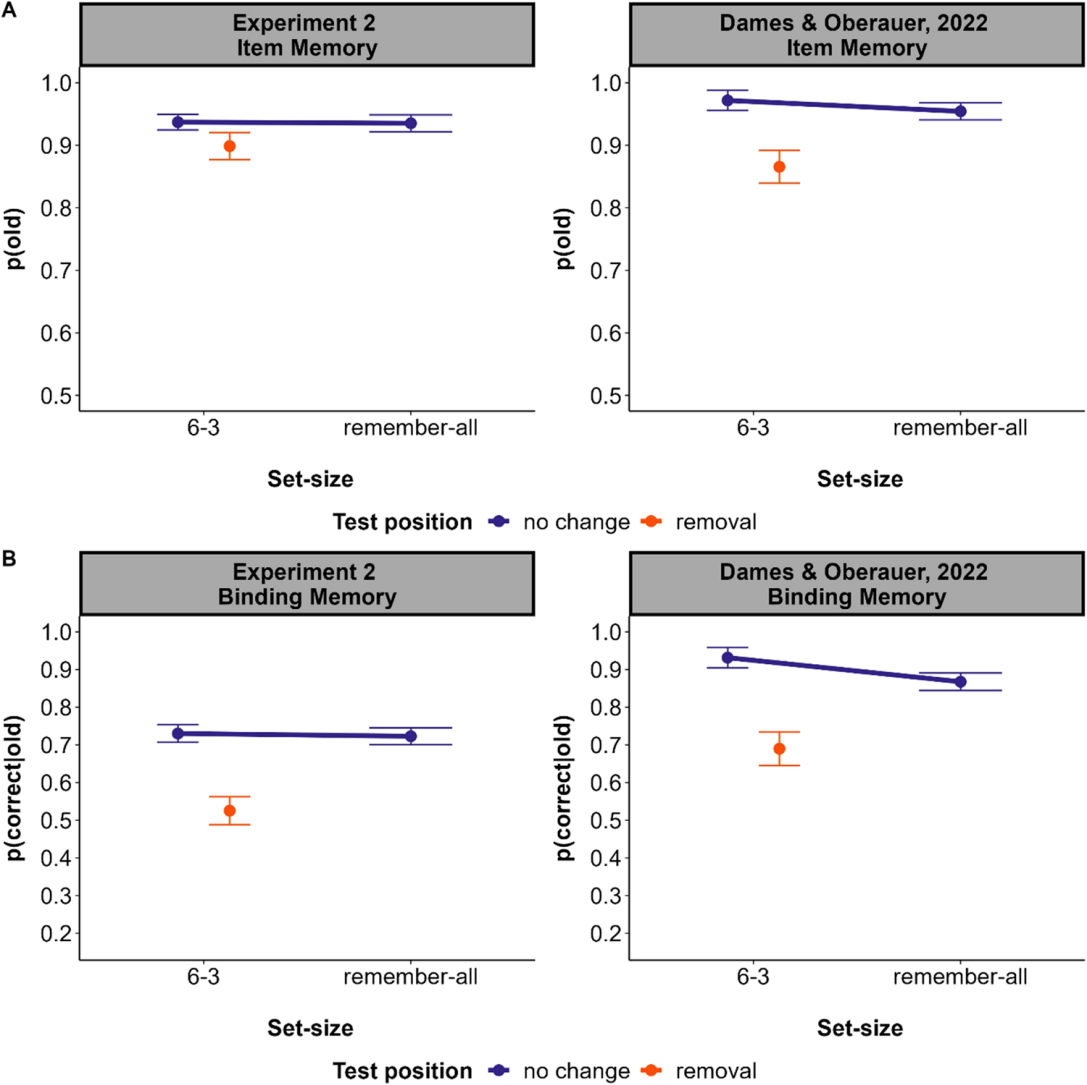
Item memory (top) and binding memory (bottom) from Experiment 2, and from Experiment 3 of Dames and Oberauer (2022) investigating immediate removal. Note. Error bars represent the 95% within-subjects confidence intervals. (Color figure online)

In this study we investigated to what extent people can selectively remove items from an already fully encoded set of items stored in WM (delayed removal). We found that although to-be-forgotten items could be still recognized (item memory), memory for to-be-forgotten item–location relations (binding memory) was substantially poorer than for to-be-remembered ones, demonstrating removal costs. Thus, we observed evidence for delayed removal. Notably, this delayed removal did not confer any benefits to the content still maintained in WM. This contrasts with earlier experiments on immediate forgetting: Forgetting items immediately after encoding encompasses forgetting costs for both binding and item memory, and benefits the remaining WM content (Dames & Oberauer, 2022). Figure 5 shows the results of the present Experiment 2 alongside of those of a comparable immediate-removal experiment from Dames and Oberauer (2022). There is a clear removal benefit of immediate removal (i.e., better performance with set size 6–3 than remember-all), which is absent with delayed removal. This contrast has already been shown in Oberauer (2018) and has led to the suspicion that delayed removal is not possible. The novel contribution of the present experiments is to demonstrate that delayed removal is effective, as it causes a removal cost on binding memory.

Delayed removal is effective with respect to forgetting information, but it fails to yield an advantage for the remaining WM contents. Hence, removing three of six items from WM after all six items have been encoding does not create a situation equivalent to only encoding three items. Considering the two best-supported families of theories of WM capacity, resource theories and interference theories (Oberauer et al., 2016), we next discuss implications of this result for our understanding of how our WM capacity is managed.

Resource theories rest on the assumption that WM has a limited resource that needs to be allocated to representations of the to-be-remembered items to maintain them. When some items from the current memory set are removed, the resource share that has been allocated to them could be re-allocated to the remaining items. Apparently, that does not improve their memorability. Should we expect that it does? That depends on how we conceptualize the maintenance resource of WM. One possibility is to think of the resource as conveying strength to a memory representation. A stronger memory trace is more likely to be recalled. This is how, for instance, the limited resource in ACT-R is implemented (Anderson et al., 1996). With this concept, it should be possible to re-allocate the resource from a removed item to the remaining to-be-remembered items to increase their strength, improving their chance of being remembered.

An alternative concept of WM resource is based on the sample-size theory (Schneegans et al., 2020; Sewell et al., 2014). Here, the resource is a limited number of units that can be assigned items in WM and code information with a certain precision. The more units are assigned to an item, the more precisely it can be represented because the precision of all units together increases with the number of units coding the same information, in the same way as the estimation of a population mean becomes more precise as the sample size of a study increases. In this conception of a resource, the resource allocated to an item determines how many units are recruited to represent independent samples of the same information from the stimulus. The individual samples must be independent so that their noise is averaged out to yield the increase of precision from a larger sample size. When an item is removed from WM, the units that have been allocated to it can be assigned to other, to-be-remembered items. However, at that time, the stimuli are no longer present, so that these units cannot represent further independent samples of the stimulus information. They could only receive copies of the information in units that have already been allocated to an item. Copying information from one unit to another does not add to the precision of the overall representation (in the same way as copying data from subjects does not improve the precision of measurement of a mean). In other words, re-allocating resources to a to-be-remembered item at a time when stimulus information is no longer available cannot create further information about that item, and thereby cannot improve the precision with which it is represented. Hence, the sample-size concept of a memory resource provides an explanation for why delayed removal does not lead to a removal benefit for the remaining items in WM. By contrast, immediate removal could lead to a benefit because the units that are freed up by removing a just-encoded item could be used to encode subsequently presented items.

From the perspective of interference theories of WM capacity, removing some of the items of a set should reduce interference for the remaining items. In one interference model, SOB-CS (Oberauer et al., 2012), removal of no-longer relevant information has been implemented as the unbinding of items from their contexts. In the present experiments, the items are the words, and the contexts are their boxes. Unbinding operates through Hebbian anti-learning, which is Hebbian learning with a negative learning rate. Hebbian anti-learning requires that the to-be-removed content representation and its context are activated, so that the Hebbian learning rule can be applied and reduce the associative strength between simultaneously active elements. This could cause difficulties for delayed removal: At the time a word is cued as to-be-forgotten, that word is no longer visible, and therefore must be retrieved from memory. To the extent that the representation of the word that is re-activated through retrieval is inaccurate, the unbinding mechanism removes the wrong content from WM, thereby damaging representations of to-be-remembered items, and insufficiently removing interference from to-be-forgotten items. The net effect could be little or no removal benefit. The expected removal benefit is higher for immediate removal because in that situation, the representations of content and context that were just bound together to encode the last-presented item are still active, and can be used to remove it again. Removal would consist of re-running the Hebbian learning process that encoded the items after flipping the sign of the learning rate, thereby exactly reversing the encoding process.

In sum, both resource and interference theories can explain why delayed removal is effective in reducing access to the removed representations while not improving access to the remaining to-be-remembered representations. One way in which the two accounts differ is in their expectations for updating. In resource theories, the resource share recovered from removed items could be fully assigned to new items that replace them, and memory after a few updating steps should be as good as for a condition without updating, with the same set size. By contrast, the interference account implies that delayed removal is imperfect, leaving residual representations of the to-be-removed items behind, which should interfere with not only the remaining old items but also new items that replace the removed ones. Therefore, memory after updating should be poorer than memory in a no-updating condition. Comparing performance in the updating conditions of the present Experiment 2 (in particular, 6–0, with 3 updating steps) with the remember-all condition shows that updating incurs a cost, which appears to be higher for the not-updated than the new items. A substantial cost of updating has also been observed in other studies (Frischkorn et al., 2022; Li et al., 2024). This is in line with the predictions from the interference account, though it does not rule out a resource account, because there are other possible reasons for why updating incurs a cost that could be reconciled with a resource theory of WM capacity.

To conclude: It is possible to selectively remove individual items from a set in WM after the entire set has been encoded. This does not make access to the remaining items better, but it is a prerequisite for continuously updating WM without overloading the system.

## Data Availability

All experiments were preregistered prior to data collection (on OSF, Experiment 1: https://osf.io/ae632; Experiment 2: https://osf.io/e5krc). The data as well as materials are publicly available on OSF (Dames et al., 2023: https://osf.io/dc9tq/).

## Footnotes

1 Please note, we originally preregistered to use moderately informative Cauchy priors with a scale of 0.353 and a mean of 0. However, given the wide tails of the Cauchy distribution, this leads to an overrepresentation of very small or large probabilities due to the used logit link function. Therefore, priors following a normal distribution are more appropriate for our data. We still ran all analysis with the preregistered priors, and this did not affect the conclusions that can be drawn from our results.

## Code availability

The analysis scripts are publicly available on OSF (Dames et al., 2023: https://osf.io/dc9tq/).
